# Mesenchymal stem cell therapies evidence in the treatment of irradiated salivary glands: A scoping review

**DOI:** 10.4317/jced.62242

**Published:** 2024-12-01

**Authors:** Maria Stella Moreira, Maria Emília Mota, Suely Kunimi Kubo Ariga, Graziella Chagas Jaguar, Márcia Martins Marques

**Affiliations:** 1 Department of Stomatology, A.C. Camargo Cancer Center, São Paulo, SP, Brazil; 2 Department of Stomatology, School of Dentistry, University of São Paulo, São Paulo, SP, Brazil; 3 School of Medicine, Emergency Medicine Laboratory, University of São Paulo, São Paulo, SP, Brazil; 4 AALZ, Sigmund Freud University, Vienna, Austria

## Abstract

**Background:**

Radiotherapy is one of the main treatments for head and neck cancer; however, due to its non-selectivity the glandular tissue can be affected. This scoping review aimed to identify the evidence about mesenchymal stem cell therapies for irradiated salivary gland regeneration.

**Material and Methods:**

Two independent reviewers performed a literature search in MEDLINE/PubMed, Scopus, and Web of Science. The inclusion criteria were: 1) studies evaluation regeneration of irradiated salivary glands by stem cell therapies (cell-based or cell-free), (2) *in vivo* studies.

**Results:**

The search resulted in 13 included studies. In general, both therapies demonstrated increased salivary levels, with mucin and amylase increased and structural protection of acinar cells. The cell-free therapy based on labial glands stem cell extract demonstrated a higher number of parasympathetic nerves.

**Conclusions:**

Stem cell therapies (cell-free and cell-based) appear promising strategies for recovering saliva production in patients presenting irradiation-induced hyposalivation, with positive results toward regeneration of the form and function of the glands. However, due to the scarcity and heterogenicity of these pre-clinical studies, it is not possible to indicate which is the more indicated therapy.

** Key words:**Mesenchymal stem cells, extracellular vesicles, exosomes, salivary glands, stem cell biology, hyposalivation, radiotherapy.

## Introduction

Saliva is the first digestive fluid, composed of water, electrolytes, proteins, and enzymes that play essential functions, such as the ingestion and digestion of food and protection against infections, due to the maintenance of oral pH and its antibacterial, antifungal, and antiviral properties (1). Salivary secretion is an energy-dependent process mediated by parasympathetic and sympathetic autonomic innervation. The fluid is secreted by acinar cells under stimulation of muscarinic and a1-adrenergic receptors (2). The salivary gland secretion can be impaired in some pathological conditions as undesirable side effects of cancer treatment (3,4).

The estimated global cancer cases are projected to reach 28.4 million by 2040, representing a 47% increase from 2020 (5). One of the main treatment modalities is radiotherapy; however, due to its non-selectivity, it could lead to adverse effects (6). When indicated for the treatment of head and neck tumors the glandular tissue can be affected causing damage mainly to the secretory epithelium and resident stem cells (3,4).

The treatment of hyposalivation and xerostomia is challenging and independent of these conditions’ etiology and the treatment does not usually differ (7) consisting, for example of pharmacological drugs, artificial saliva, or lasers (8). Moreover, these treatments remain palliative since they depend on remaining acini (9). To circumvent this problem, tissue engineering with re-implantation of autologous salivary gland cells, engineered artificial salivary glands, mesenchymal stem cell therapies, and gene therapy have been studied, aiming to promote more effective and permanent treatments (9,10).

Investigations on substituting or regenerating the salivary gland epithelium to recover its secretory function have demonstrated that transplantation with mesenchymal stem cells (MSCs) (cell-based therapy) can rescue the morphology and function of salivary glands damaged by irradiation (3). Furthermore, studies have demonstrated that the paracrine effects resulting from MScs are responsible for these therapeutic effects (cell-free therapy) (3,11).

However, there is no consensus about the most applicable strategy to apply for salivary gland regeneration. Thus, this scoping review aimed to identify the evidence about mesenchymal stem cell therapies for irradiated salivary gland regeneration.

## Material and Methods

The recommendations of Arksey and O’Malley’s (12) and Joanna Briggs Institute (13) were used to develop this scoping review and the Preferred Reporting Items for Systematic Reviews and Meta-analysis – Extension for scoping reviews (PRISMA-ScR) was used to report (supplementary material) (14).

-Search strategy and study selection

A literature search was conducted looking for papers published up to and including June 2024, based on the PCC questions (Population: degenerated tissues; Concept: dental mesenchymal stem cells; context: tissue regeneration). Relevant articles were searched and obtained from online databases: PubMed/MEDLINE, Web of Science, and Scopus. Keywords and Medical Subject Headings (MeSH) terms were used for searching, and Boolean operators (OR, AND) were used to combine searches. The following search was performed in MEDLINE/PubMed and adapted for the other databases: ((((salivary gland) OR (salivary glands)) AND (((radiation) OR (radiotherapy)) OR (irradiated))) AND (((((((((((stem cell) OR (stem-cell)) OR (stem cells)) OR (stem cells)) OR (stem-cells)) OR (cell-homing)) OR (cell homing)) OR (cell free)) OR (cell-free)) OR (secretome)) OR (exosome))) AND (regeneration). All identified duplicates were manually deleted. All titles and abstracts of studies found were independently assessed by two reviewers (M.E.M and M.S.M), based on the following inclusion criteria: (1) studies evaluation regeneration of irradiated salivary gland by stem cell therapies (cell-based or cell-free therapies), (2) *in vivo* studies. The language and data of the publication had no restrictions. The reviewers assessed the full text, considering the following exclusion criterion: (1) studies that focus on other regenerative therapies that do not involve stem cells. Disagreements were resolved by consensus-based discussion with a thirty researcher (M.M.M).

-Data collection and extraction

The reviewers independently extracted information of interest from all the included articles using Tables. Information extracted included: authors and year, animal and irradiation model, stem cell type, cell-free strategy, number of cells, delivery method, scaffold, and main findings.

Results

**Figure 1:**
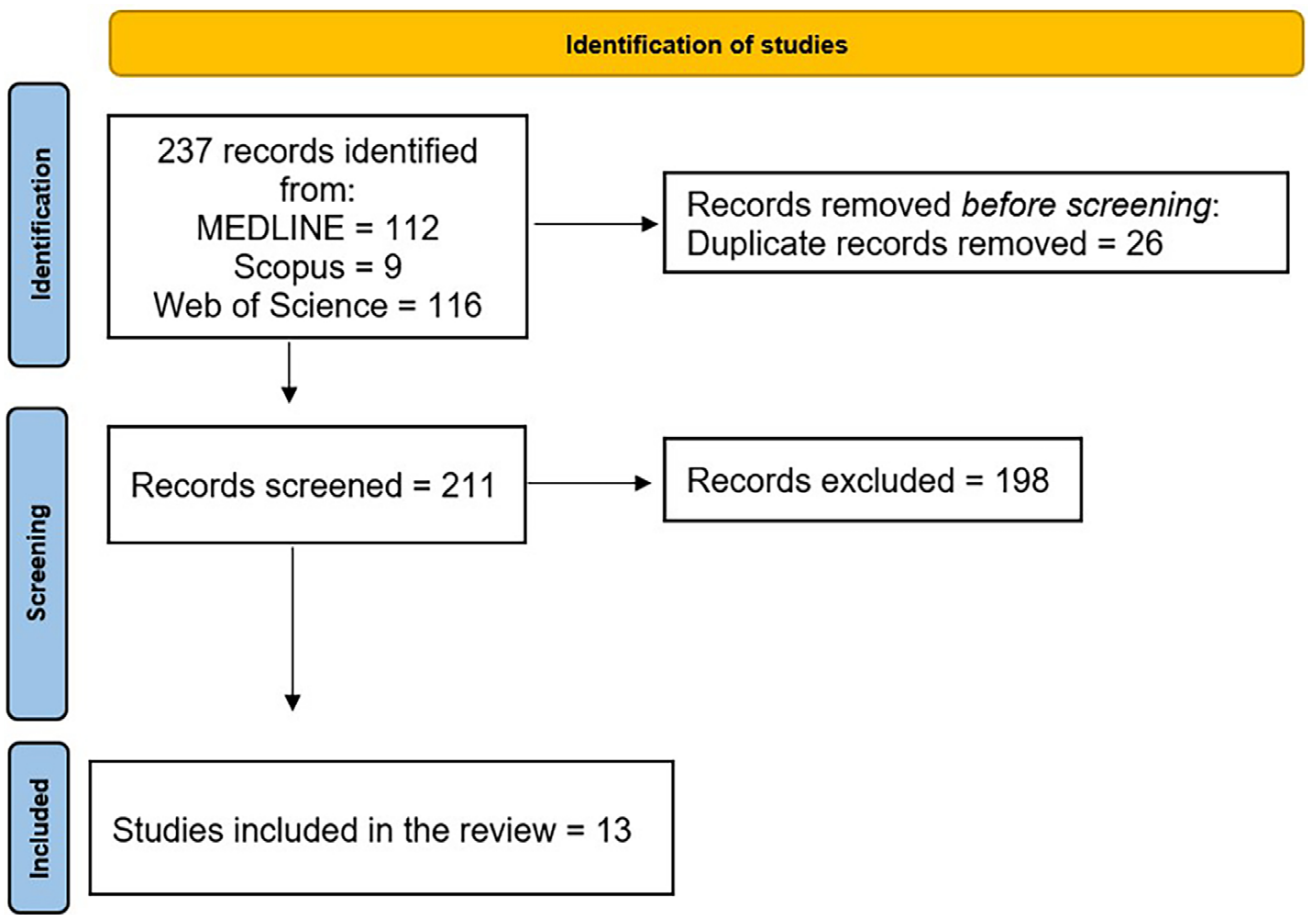
Flowchart.

Figure 1 presents the flowchart summarizing the process of study selection. For screening studies, of the initial 237 potentially relevant articles identified by the search, 26 duplicates were excluded and 211 were considered eligible. Of these, 198 (93.8.3%) were excluded based on the inclusion and exclusion criteria, and finally, 13 articles were selected for this review.

-Collected data 

 Table 1 describes the main information from pre-clinical studies of cell-based therapies for regeneration of irradiated salivary glands. Most studies used orthotopic experimental models with mice (10,15-18). Rats (19) and pigs were also used (20,21). The animals had their salivary gland irradiated with doses between 5Gy and 20Gy (10,15-21). Regarding the stem cell type used for salivary gland regeneration, adipose tissue was the predominant source (10,16,17,20). Stem cells isolated from bone marrow (15,19), salivary gland (18) and gingiva (21) were also used. Only 3 authors reported the use of scaffolds, and the strategies consisted of: adipose tissue-derived mesenchymal stem cells (AdMSCs) combined with heterologous platelet-rich fibrin (PRF) and injected through the capsule of both submandibular glands (16). An AdMSCs-loaded hydrogel of decellularized porcine small intestinal was injected directly into both submaxillary glands (17), and bone marrow stem cells suspended in platelet-rich plasma (PRP), were applied also by intraglandular injection (19). Cells (1x103 to 1x107) (10,15-21) were administered systemically from the tail vein (10,15) and lateral saphenous vein of the hind leg (21) or intraglandular with injections (16-20). Six studies observed an increase in salivary flow rate after a period between 8 and 24 weeks (10,15-17,21). The anti-apoptotic effect was observed in 5 studies (10,15-17,21), in addition, anti-oxidative activity was also identified (20).

An increase in the surface area and quantity of acinar cells was reported (10,16,19-21), in addition to the reduction in atrophy (10) and higher levels of production of mucin and amylase (10,16,20). Moreover, the authors described a greater number of salivary epithelial cells (AQP-5), endothelial (CD31), myoepithelial (α-SMA), and stem cells (sca-1 and c-kit) (15,17). A higher expression of the transforming growth factor β1 (TGF- β1) gene was also observed (19), as well as an increased level of blood vessel formation (15) and again cell proliferation (15,21).

 Table 2 displays the main data information from the 5 included studies of cell-free therapies for regeneration of irradiated salivary glands. Four studies were performed in mice (9,22-24) and 1 rat (25), which had their glands irradiated with doses between 5Gy to 27.5Gy (9,22-25). The studies used MSCs derived-secretome of adipose tissue, extract of labial glands, extracellular vesicles of dental pulp, exosomes of urine and conditioned medium derived from human exfoliated deciduous teeth (9,22-25). All studies performed systemic administration via the tail vein, without scaffolds (9,22-25).

The improvement in salivary flow rate was observed among the main findings, present in 3 out the 5 studies, at 2 and 16 weeks after starting the cell-free therapies (9,22). Increased levels of amylase, mucin, and epithelial growth factor (EGF) were also reported in 1 study, and there was also integrity of the glandular structure (22). Salivary epithelial cells (AQP-5) increased in number in 2 studies (22,23), in addition, a decrease of apoptosis leading to protection of endothelial (CD31), myoepithelial (α-SMA) (22), and SG progenitor cells (c-Kit) (22,25) against radiation damage were observed. Moreover, increase in salivary flow rate along with higher numbers of acinar cells, blood vessels, and parasympathetic nerves were reported in 1 study (9). Furthermore, a reduction of the expression of the senescence genes and inflammatory cytokines (23), upregulation of Wnt3a, GSK3B and Axin (25) and increase of the expression of antioxidant enzymes (24) were also reported.

## Discussion

Radiotherapy is the treatment for approximately 70% of cases of head and neck cancer. Although effective, this therapy can result in hyposalivation due to the exposure of the salivary glands to radiation (26), with a loss of 50% to 60% of saliva in the first week (19). The continuous progress in craniofacial tissue engineering has shown that stem cell therapies can constitute a more efficient and precise treatment for damage to salivary glands caused by radiation. The strategies addressed in this review consist of stem cell therapies as treatment for radiation-induced xerostomia in pre-clinical studies.

MSCs are identified in many human tissues, such as bone marrow, adipose tissue, amniotic fluid and membrane, dental tissues, and salivary glands, among others (27). For treating radiation-induced xerostomia with cell-based therapies, adipose tissue stem cells were the most used (10,16,17,20). According to studies adipose tissue cells are relatively less affected by the donor’s age (10) and contain a higher density of MSCS than bone marrow (10,17), especially when collected from liposuctions (28). However, reports have indicated that local anesthetics used can negatively impact the quantity and viability of adipose tissue cells. Furthermore, apoptosis of most cells is reported a few days after transplantation (28). The use of stem cells derived from dental pulp, labial minor salivary glands and gingiva was also considered an important source of MSCs for salivary gland regeneration. These cells are also easy to obtain (9,18,21,23,24), especially those from dental pulp, as they can be collected from dental tissues that are usually discarded. Dental pulp stem cells also have immunomodulatory and anti-inflammatory capacity and low risk of immunological rejection (29-31).

Autologous or allogeneic stem cell transplantation from different sources has shown positive results in protection against cellular damage caused by irradiation (10,15-21). In general, studies have demonstrated restoration of gland morphology, regenerating, and preserving acini, increasing blood vessels, cell proliferation, and reducing fibrosis, in addition to recovering function, with improvement in salivary flow rates in up to 24 weeks (10,15-21). However, cell transplantation depends on sufficient tissue for cell expansion and long culture times to obtain enough cell numbers (9). This appears even more complex in the clinical setting, one clinical study, for example, required 25x106 per gland (32). The regenerative potential of stem cells also depends on the age of the donor (3). Another important point is the decline in graft viability, which demonstrates a low survival rate and impaired differentiation capacity of MSCs transplanted *in vivo* (33).

Several animal models were investigated in the preclinical studies (9,10-25). However, there are some challenges to irradiating orthotopic models. High-precision equipment in defining the target volume is necessary for small animals (34). Furthermore, for dose tolerance, fractional radiation dose adjustment is required (35). Miniature pigs appear to be a better animal model for salivary gland disease study, due to their glandular structure and ductal system like those of humans (20).

In 2 studies, administration of cell-based therapies was performed from the tail vein (10,15). However, the most recent studies performed intraglandular injections (17-20). Clinical studies also executed stem cell application to the glands, using ultrasound (32,36). Both vias lead to good results in glandular tissues; however, some authors point out that fewer doses of intraglandular injection are needed for gland repair (37) and that intravascular injections of cells can result in thromboembolic complications (9). The studies assessing the cell-free therapies have primarily focused on tail vein administration in mice and rats (9,22-25). The intraglandular application has not been evaluated yet, however, this approach is more like clinical applications and holds the potential to improve the results.

Studies demonstrated that paracrine effects are the main mechanism of tissue remodeling; therefore, cell-free therapies are considered a therapeutic strategy for salivary gland engineering (3,10). MSCs secret trophic factors and cytokines with anti-inflammatory, immunoregulatory, and angiogenic properties through secretomes (10,24). Extracellular vesicles (microvesicles and exosomes) are responsible for transporting cellular load, such as growth factors, cytokines, and RNAs (22,23). In addition, previous studies reported that MSCs products present lower histocompatibility antigens than intact cells, in addition to being more viable for clinical application, especially due to cell storage limitations (38). However, the clinical application of exosome is limited, as there is still no standardization of the isolation, purification and identification of exosomes (39).

Hyposalivation results from acute and late effects of radiation. Acute effects stem from damage to the plasma membrane of secretory cells. On the other hand, late effects include apoptosis of progenitor cells and reduced blood flow, growth factors, cytokines, and extracellular and anti-apoptotic proteins (36). Here, we observed that cell-free therapies were also able to improve glandular function and morphology, especially with the improvement of salivary flow rates within 16 weeks, decrease of apoptosis, and increase in the number of acini (9,22,23,25). Furthermore, a higher density of parasympathetic nerves was observed after injections of extract of labial glands stem cells in the tail vein of mice (9). This is especially relevant because parasympathetic innervation is responsible for the main part of salivary flow (40). Thus, without functional innervation and in the absence of factors that maintain neuronal-epithelial communication, progenitor cell regeneration does not occur (41).

Some authors demonstrated that salivary gland stem cells can survive, remaining dormant after irradiation. A subculture of these colonies was performed, confirming this finding once intense multipotency was observed depending on the radiation dose and age of the cells (42). The evaluated studies demonstrated that MSC transplantation increased the expression of stem cell markers (Sc1-a and c-kit), highlighting the possibility of reactivation of these cells (15,17). Furthermore, therapies with secretome and exosomes demonstrated protection and an increase in c-kit cells (22,25).

Tissue engineering approaches are based not only on the use of cells, on but also bioactive molecules and scaffolds (43). Most studies did not use scaffolds for glandular regeneration; the use of natural scaffolds based on decellularized extracellular matrix hydrogel and platelet aggregate (PRF and PRP) was investigated only in 3 cell-based therapy studies (16,17,19). The selection of an appropriate scaffold assists in stem cell survival and retention. InjecTable decellularized extracellular hydrogels are a highly promising option for therapeutic applications due to their target-specific minimally invasive delivery and their capability to fill tissue defects. Choi *et al*. observed that the group used the porcine small intestinal submucosa hydrogel associated with adipose tissue stem cells, finding a decrease in the number of apoptotic cells, compared to isolated cells (17). Furthermore, the use of PRP and PRF is associated with growth factors and cytokines, including TGFβ, VEGF, and fibroblast growth factor-2 (16,17) It is demonstrated that these factors aid in neovascularization and increase cellular survival of the glands (16,17).

The use of scaffolds associated with cell-free therapies was not observed in this review, however, studies demonstrated their importance, mainly due to the possibility of acting in the controlled release of factors in the tissues (44). Furthermore, cell-free therapies using scaffolds have demonstrated better results in angiogenesis, neurogenesis, tubulogenesis, proliferation and cell viability (44-47).

Cell-free and cell-based therapies appear promising strategies for recovering saliva production in patients presenting irradiation-induced hyposalivation. The studies here evaluated showed mostly positive results toward the regeneration of form and function of the salivary glands as result of both techniques. It is important to highlight that therapies deserve to be further tested in more pre-clinical studies to determine not only which would be the best therapy (cell-free or cell-based) but mostly to indicate the best source of MCS cells and scaffolds to be applied for reaching the highest efficiency with lesser damage to the patient.

## Figures and Tables

**Table 1:** Cell-based therapies/pre-clinical studies.

Author, year	Animal model	Irradiation model	Stem Cell type	Number of cells	Delivery method	Scaffold	Main findings
Sumita 2011	Mice	18Gy	Bone marrow	1x10^7^	Tail vein injection	NR	-Salivary output was increased at weeks 8 and 24 post-irradiation - Lower apoptotic activity at 24 weeks -Increased level of blood vessel formation and cell proliferation at 24 weeks -Expression of stem cell markers (Sca-1 or c-kit), absent in irradiated non-transplanted glands - Higher regeneration of acinar cells
Lim 2013	Mice	15Gy	Adipose tissue	1x10^6^	Tail vein injection	NR	-Improved salivary flow rates at 12 weeks after irradiation -Fewer damaged and atrophied acinar cells - Less periductal and perivascular fibrosis - Greater number of acini -Higher mucin and amylase production -Fewer apoptotic cells - Higher proliferation indices at weeks
Pringle 2016	Mice	5Gy	Salivary gland (salispheres)	5x10^2^. 5x10^3^, 5x10^4 ^per gland	Intraglandular injection	NR	-Increase of salivary flow at 2 and 3 months - Enhanced salisphere formation compared to culture generated from the irradiated group
Wang 2017	Miniature pig	20 Gy	Adipose tissue	4x10^6 ^per gland	Intraglandular injection	NR	-Increase of salivary flow rate - More functional acinar cells preserved - More amylase production
Wang 2017	Mice	18 Gy	Adipose tissue	2x10^5 ^per gland	Intraglandular injection	Rabbit heterologous platelet-rich fibrin extract	-Increased the salivary flow rate at 12 weeks post-transplantation -More preserved structures and greater number of acini -Higher number of mucopolysaccharide-containing acinar cells - Higher number of microvessels - No significant ultramicrostructural damage -Fewer damaged and atrophied acinar cells -Higher α-amylase -Decreased apoptotic and increased proliferative activity
Choi 2018	Mice	15 Gy	Adipose tissue	1x10^5^/20µl of SIS	Intraglandular injection	porcine small intestinal submucosa hydrogel (SIS)	- Higher salivary flow rate at 16 weeks after treatment - Less periductal fibrosis -More mucin-producing acini -Greater number of salivary epithelial cells (AQP-5), SG progenitor cells (c-Kit), endothelial cells (CD31) and myoepithelial cells (α-SMA) - Anti-apoptotic and anti-oxidative effects
Mohamed 2022	Rats	6 Gy	Bone marrow	0,5 × 10^5^	Intraglandular injection	platelet-rich plasma	-Increase in the surface area of acini - Increase in TGF- β1 gene expression
Zayed 2024	Pigs	15 Gy	Gingiva	1 x 10^6^	Lateral saphenous vein of the hind leg injection	NR	-Restoration of acinar and tubular structures -Increase in cell proliferation -Reduction in apoptotic activity

Abbreviations: Gy = Gray / NR = Not reported / AQP-5 = Aquaporin 5 / α-SMA = alpha-smooth muscle actin

**Table 2:** Cell-free therapies/preclinical studies.

Author, year	Animal model	Irradiation model	Stem cell type and cell-free strategy	Delivery method	Scaffold	Main findings
An 2015	Mice	15Gy	Secretome of adipose tissue	Tail vein	NR	-Improve of improved the ratio of salivary flow rates at 16 weeks -Increased levels of amylase, mucin and EGF -Microscopic structural integrity of the salivary gland - Salivary epithelial (AQP-5), endothelial (CD31), myoepithelial (α-SMA) and SG progenitor cells (c-Kit) were protected -Decrease of apoptosis
Su 2020	Mice	13Gy	Extract of labial glands	Tail vein	NR	-50% to 60% higher salivary flow rate at 8 weeks - Higher numbers of acinar cells, blood vessels, and parasympathetic nerves and cell proliferation rates
Dong 2021	Mice	25Gy	Extracellular vesicle of dental pulp	Tail vein	NR	- Higher AQP5 expression levels - Reduction the expression of the senescence genes and inflammatory cytokines
Xiao 2022	Rats	27.5Gy	Exosome of urine	Tail vein	NR	-Decrease of expression of a-SMA -Increase of c-kit - Upregulate of Wnt3a, GSK3B, Axin
Kano 2023	Mice	5 Gy	Conditioned medium derived from stem cells from human exfoliated deciduous teeth	Tail vein	NR	-Activation of antioxidant enzyme genes in the target tissue - Improve of salivary flow rate at 2 weeks

Abbreviations: Gy = Gray / NR = Not reported

## Data Availability

The datasets used and/or analyzed during the current study are available from the corresponding author.
